# Smog Nitrogen and the Rapid Acidification of Forest Soil, San Bernardino Mountains, Southern California

**DOI:** 10.1100/tsw.2007.74

**Published:** 2007-03-21

**Authors:** Yvonne A. Wood, Mark Fenn, Thomas Meixner, Peter J. Shouse, Joan Breiner, Edith Allen, Laosheng Wu

**Affiliations:** ^1^ Department of Environmental Sciences, University of California, Riverside, CA, USA; ^2^ USDA Forest Service, Pacific Southwest Research Station, Riverside, CA, USA; ^3^ Department of Hydrology and Water Resources, University of Arizona, Tucson, AZ, USA; ^4^ USDA-ARS George E. Brown, Jr. Salinity Laboratory, Riverside, CA, USA; ^5^ Department of Botany and Plant Sciences, University of California, Riverside, CA, USA

**Keywords:** Soil pH, nitrogen, soil hydrology, air pollution, forest soils, stone lines, geochemistry, landscape-atmosphere interactions

## Abstract

We report the rapid acidification of forest soils in the San Bernardino Mountains of southern California. After 30 years, soil to a depth of 25 cm has decreased from a pH (measured in 0.01 M CaCl_2_) of 4.8 to 3.1. At the 50-cm depth, it has changed from a pH of 4.8 to 4.2. We attribute this rapid change in soil reactivity to very high rates of anthropogenic atmospheric nitrogen (N) added to the soil surface (72 kg ha–1 year–1) from wet, dry, and fog deposition under a Mediterranean climate. Our research suggests that a soil textural discontinuity, related to a buried ancient landsurface, contributes to this rapid acidification by controlling the spatial and temporal movement of precipitation into the landsurface. As a result, the depth to which dissolved anthropogenic N as nitrate (NO_3_) is leached early in the winter wet season is limited to within the top ~130 cm of soil where it accumulates and increases soil acidity.

## INTRODUCTION

Soil acidification is a natural process that operates over many thousands of years across forest wildland soils. However, forest soils near urban regions where emissions of anthropogenic nitrogen (N) are high have shown accelerated acidification that is detectable within decades. In this work, we consider the rate of change of soil reactivity in the San Bernardino Mountains located in southern California. Across these mountains, several permanent research sites have been established for more than 3 decades[1]. Limited soil pH data (from the years of 1975 and 1993) are available from this long-standing research effort[2,3]. By comparing the pH of soils we sampled in 2003 and 2004 to these historic data, we consider the change in forest soil reactivity over 3 decades under conditions with and without high rates of smog N additions.

We selected two of these permanent research sites[1] for our study of change in forest soil reactivity. One of these sites, Camp Paivika (CP), receives the highest rate of atmospheric anthropogenic (smog) N

additions (72 kg ha$^{-1}$ year$^{-1}$) measured in the U.S.[4,5] and served as our site "with high rates of smog N additions". Soil pH to a depth of 3 m was compared between CP and Holcomb Valley (HV). Holcomb Valley, located in a "smog shadow" caused by the topography of the mountain massif, has low levels of atmospheric N deposition (6 kg ha$^{-1}$ year-1[5]) and served as our site "without high rates of smog N additions".

During soil sampling at the CP and HV sites for this project, we observed the consistent presence of subsurface stone lines between the 40- to 60-cm and the 130- to 170-cm depths (Fig. 1). A stone line is a three-dimensional subsurface layer of stones, often found associated with buried paleosols[6]. The observed stone lines and underlying highly weathered bedrock indicate the presence of an ancient landsurface, widespread throughout the San Bernardino Mountains, which has been uplifted by tectonic activity along the San Andreas Fault[7]. Beneath the stone lines, soil texture and soil color abruptly change in relation to the overlying soil. We hypothesized that these stone lines and associated soil textural discontinuity are important to subsurface hydrologic processes through control of the spatial and temporal movement of soil water. In turn, this should affect the rate of soil acidification through control of the movement of $NO_3$ carried as a dissolved solute.

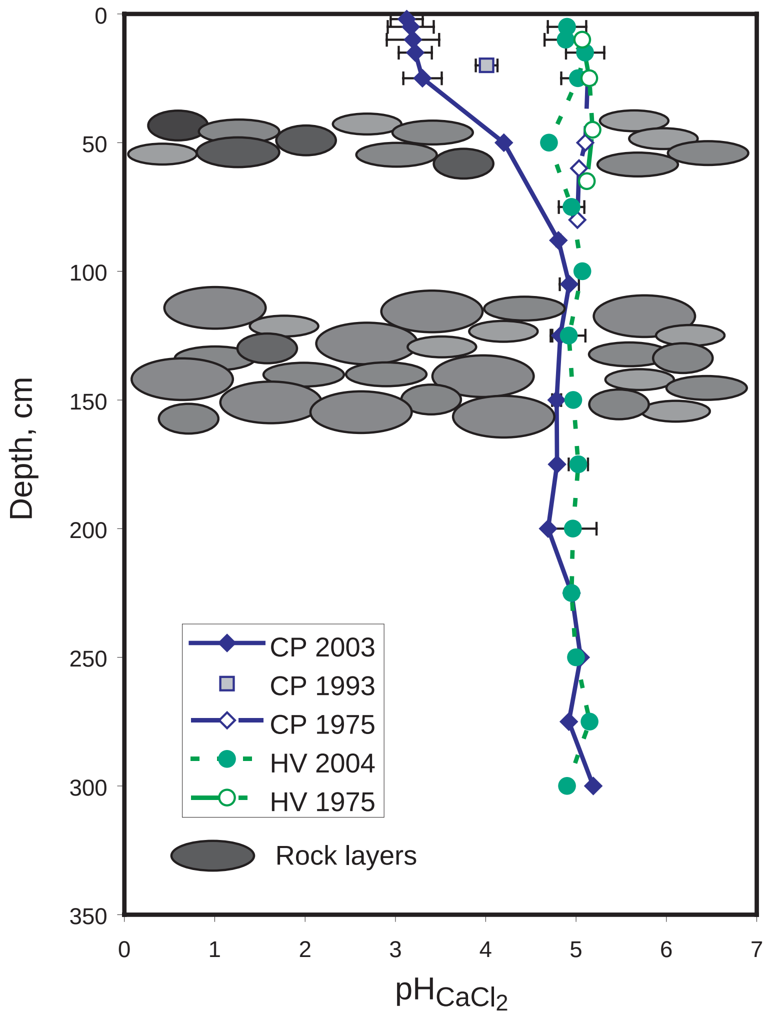

**FIGURE 1.** Soil pH with depth to 300 cm measured during 1975, 1993, 2003, and 2004 at San Bernardino Mountain forest sites with buried stone lines. HV = Holcomb Valley site where atmospheric anthropogenic N additions are minor (6 kg ha$^{-1}$ year$^{-1}$). CP = Camp Paivika site where atmospheric anthropogenic N additions are the highest reported in the U.S. (72 kg ha$^{-1}$ year$^{-1}$).

## METHODS

Soil samples (approximately 250 g each) were collected using a 6.35-cm-diameter bucket auger to hand drill cores. Soils were sampled intensively within the near surface (2-, 5-, 10-, 15-, and 25-cm depths) and then at every 25-cm-depth increment until fresh bedrock prevented deeper coring. These soil samples were analyzed in the laboratory to measure pH in 0.01 $CaCl_2$ using a glass electrode[8], and soil Cl and soil $NO_3$ concentrations using standard methods for our laboratory[9]. Historical pH values that had been measured in 1:1 soil:distilled water ($pH_{H2O}$)[2] were corrected to $pH_{CaCl2}$ values based on a correlation equation determined using soils from our study sites. Our values closely matched other reported corrections[10] between measured values of $pH_{H2O}$ and $pH_{CaCl2}$.

The temporal and spatial control of soil water movement by subsurface stone lines was studied at two of the permanent sites: CP, where buried stone lines are present and Barton Flats (BF), where the stone lines are absent. Extensive sampling to determine soil water to at least the 150-cm depth was conducted over a 2-year period using (1) monthly real-time soil moisture readings taken with a CPN 503DR neutron probe (Campbell Pacific Nuclear International, Martinez, California) and (2) annual measurements of soil chloride (Cl) made at the end of the dry season, as a conservative tracer of soil water movement[11].

## RESULTS AND CONCLUSIONS

### Addition of Atmospheric N and Soil Acidification

Soil $pH_{CaCl2}$ values measured in the fall of 2003 at CP show a dramatic change when compared to values measured at the same site during the falls of 1975 and 1993. Soil $pH_{CaCl2}$ dropped a total of 1.7 units of $pH_{CaCl2}$ within 3 decades (Fig. 1). The measured pH was observed to decrease at an increasing rate with the passage of time. A decrease of 0.8 unit of $pH_{CaCl2}$ occurred in the first 2 decades. An additional 0.8 unit of $pH_{CaCl2}$ change occurred during the 3$^{rd}$ decade.

We observed the measured $pH_{CaCl2}$ values to be directly related to the $NO_3$ concentration measured in the soil (Fig. 2), indicating that the addition of anthropogenic N is responsible for this rapid acidification. At HV, the same physical control (subsurface stones lines) on soil water and solutes movement is found (see below). However, with low atmospheric N additions (6 kg $ha^{-1}$ $year^{-1}$[5]), soil $NO_3$ concentrations (Fig. 2) are low and limited change in soil pH was seen over the studied 3 decades (Fig. 1).

### Control of Subsurface Hydrology and Translocation of Solutes by Buried Stone Lines

The buried ancient landsurface at the CP site strongly influences hill-slope subsurface hydrology. Neutron probe measurements, taken monthly over a 2-year period to a depth of 350 cm (Fig. 3 and 4), show the dramatic control of soil water, both spatially and temporally, by the buried stone lines at CP compared to BF, where stone lines are absent. At CP, early wet season soil water does not move laterally below the depth of 140 cm (Fig. 3). Instead, our study suggests that soil water moves downslope at CP within the region of the stone lines. In comparison, at BF, our neutron probe measurements show that soil water infiltrates uniformly, wetting consecutively from the surface to lower depths as the wet season progresses (Fig. 4).

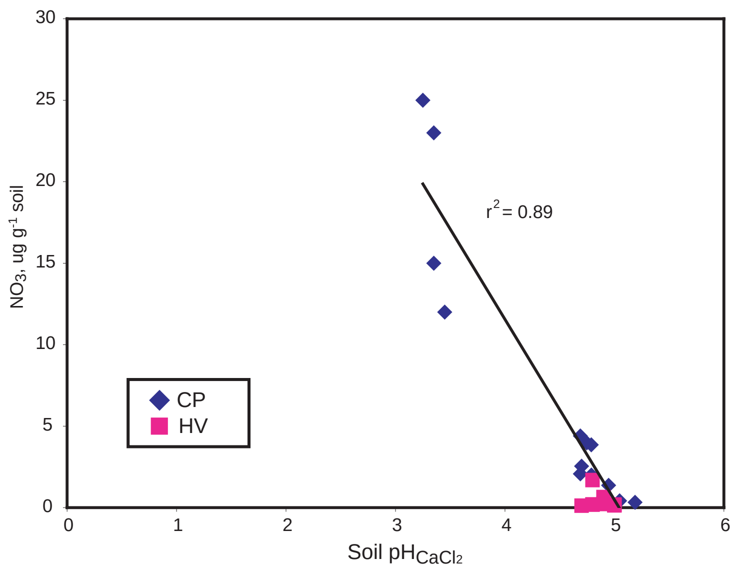

**FIGURE 2**. Soil pH in relation to soil nitrate ($NO_3$). Measurements were taken with depth during the Fall of 2003 and 2004 at CP and HV.

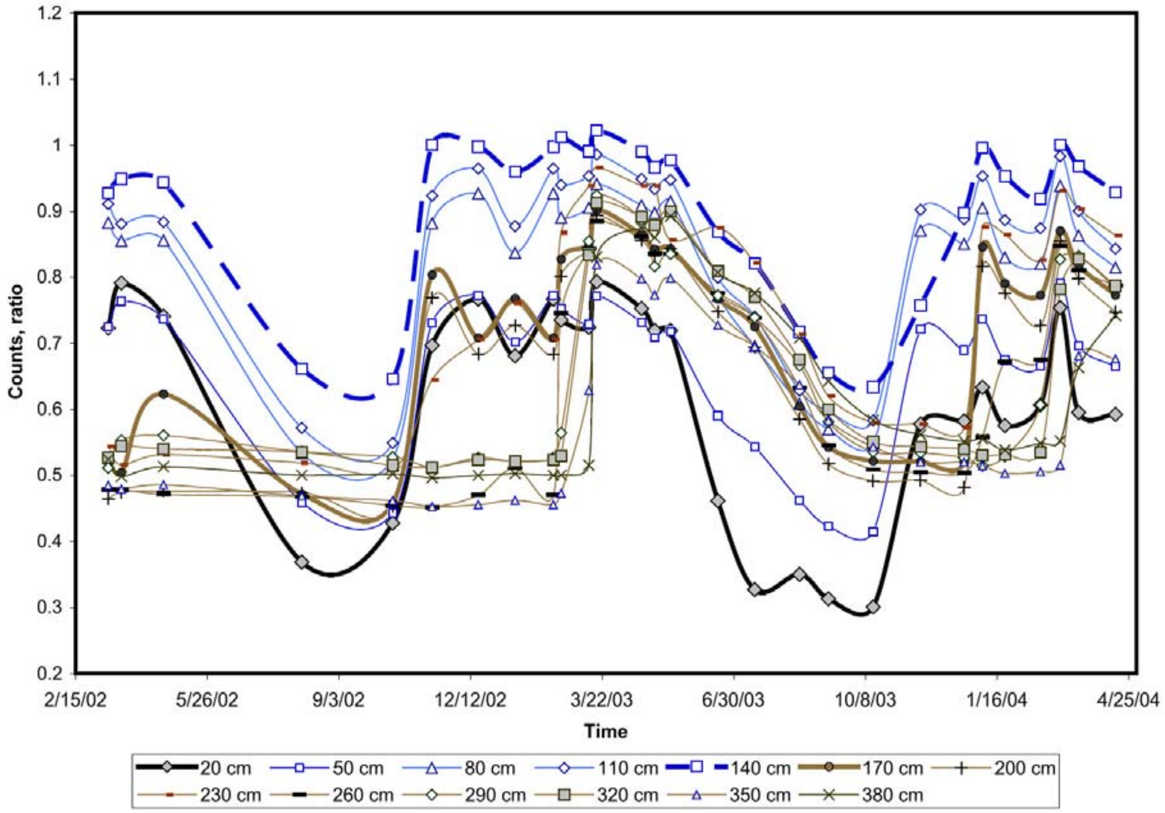

**FIGURE 3.** Neutron probe measurements taken monthly over a two year period at the CP site. Here, where subsurface stone lines occur, soil water patterns for the 140 cm depth and above are disjunct temporally from those at depths below 140 cm. (Measured soil moisture increases with increasing count ratio.)

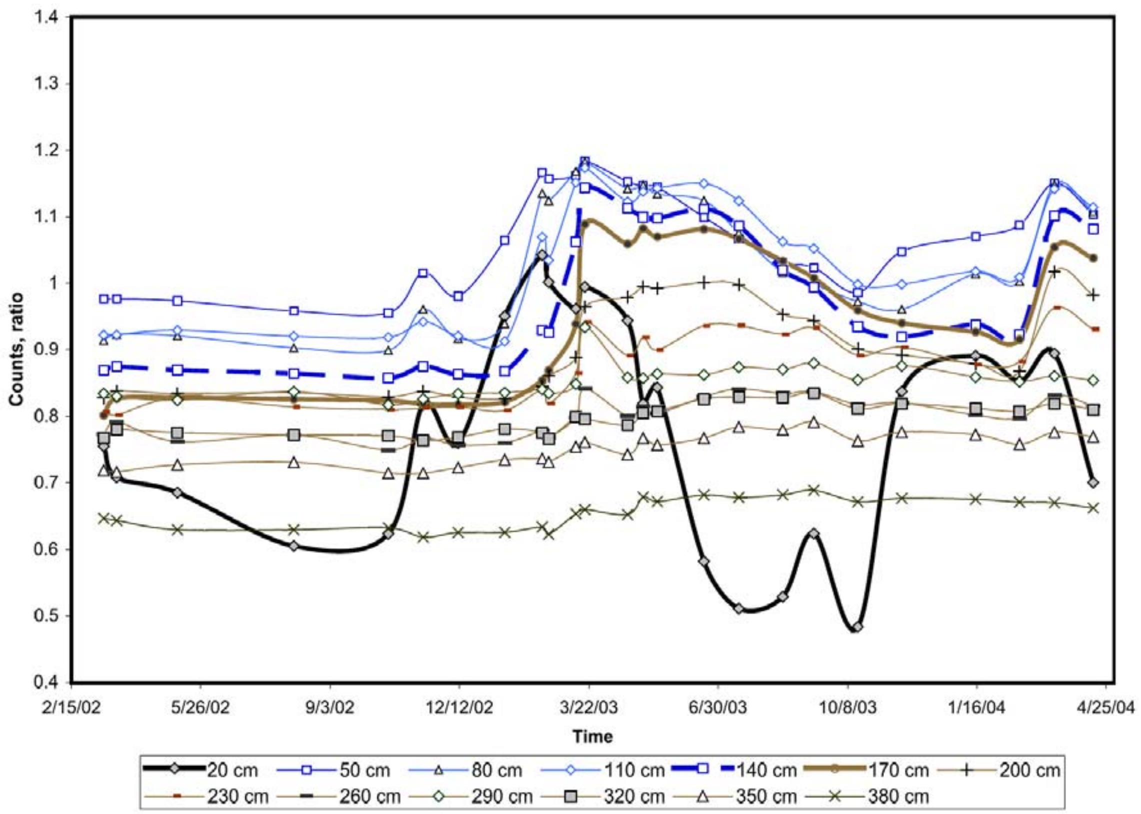

**FIGURE 4.** Neutron probe measurements taken monthly over a two year period at the BF site. Here, where subsurface stone lines are absent, the soil wets consecutively from the surface to depth with time after the wet season begins (usually in November). (Measured soil moisture increases with increasing count ratio.)

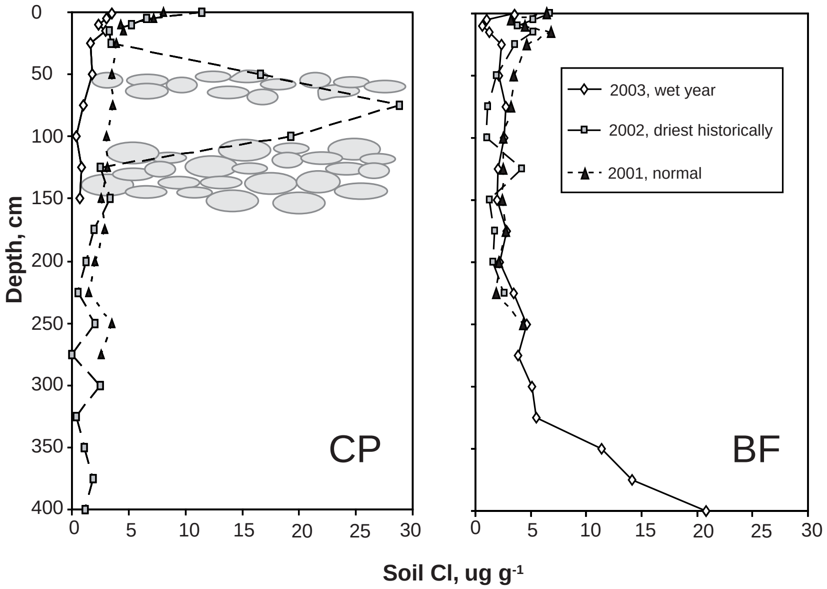

**FIGURE 5.** Patterns of soil Cl movement compared between CP, where stone lines are found, and BF, where no stone lines are found. The high concentration of soil Cl within the 25 to 100-cm soil depths at the end of the drought year of 2001-2002 at CP reflect the strong control of soil water by the stone lines.

By controlling the patterns of infiltration of precipitation, subsurface stone lines also control the patterns of leaching of soluble atmospheric additions, such as anthropogenic N as $NO_3$, from the soil surface to deeper depths. Soil cores to fresh bedrock collected at the end of the hot, dry summer period for three consecutive years (Fig. 5) highlight the dramatic difference between the movement of the soluble anion Cl within the soils at the CP (with stone lines) and BF (without stone lines) sites. The high concentration of Cl in the surface 125 cm of soil at CP during the very dry year of 2001–2002 suggests a similar pattern will occur for the addition of anthropogenic N as $NO_3$, an anion that moves through the soil nearly identical to Cl.

Thus, our data suggest that when subsurface stone lines are present, the depth of early season infiltration at CP is limited, translocating the large surface additions of anthropogenic N as $NO_3$ no deeper than the 80- to 120-cm soil depths. Under these conditions, the upper ~100 cm of soil at CP, the high N deposition site, is rapidly acidifying (Fig. 1). In contrast, at HV, the soil $pH_{CaCl2}$ has changed little during 3 decades, even though subsurface stone lines will also limit the depth of soil water and solute movement. Here, low additions of atmospheric N do not provide sufficient $NO_3$ to affect soil acidity (Fig. 2).

## ACKNOWLEDGMENTS

This research relied on the field and laboratory help of several Department of Environmental and Soil Sciences (University of California, Riverside) students and staff. The efforts of all are gratefully acknowledged. This research program was funded by The USEPA Science to Achieve Results (STAR) program (Grant No. R-82890101-0; Thomas Meixner, PI) and partially funded by NSF grant DEB 04-21530.
